# 

**Published:** 1908-07-25

**Authors:** 


### BOOKS RECEIVED.

Eliot Boothroyd.
"Low's Handbook to the Charities of London, 1908."

W. Heinemann.

"The Natural History of Cancer." By W. Roger Williams, F.R.C.S.

J. K. Lewis.
Catalogue of Lewis's Circulating Library.

John Murray.
"The Solar System." By Professor Chas. Lane.
"Climate." By Professor Robt. de Courcy Ward.

J. and A. Churchill.

"A Manual of Midwifery." 2nd edition. By T. Watts Eden, M.D.

"Ophthalmic Surgery and Medicine." By Walter H. H. Jessop, M.A., M.B.

Hy. Frowde and Hodder and Stoughton.

(The Oxford Medical Publications.)

"A System of Syphilis." Vol. I. Edited by D'Arcy Power, M.A., M.B., F.R.C.S., and J. Keogh Murphy, M.C., F.R.C.S.

(*Corrected Notice.*)

**This Coupon must accompany manuscript or contributions intended for The Hospital.**

